# Evaluation of Results of Open Reduction and Internal Fixation (ORIF) of Fracture of Distal End of Femur with Intra-Articular Extension

**DOI:** 10.5704/MOJ.2111.012

**Published:** 2021-11

**Authors:** TK Amin, I Patel, MJ Patel, MM Kazi, K Kachhad, DR Modi

**Affiliations:** Department of Orthopaedics, Smt NHL Municipal Medical College, Ahmedabad, India

**Keywords:** intra-articular, distal femur, internal fixation, plating

## Abstract

**Introduction:**

Fractures of the distal femur account for 0.4% of all fractures. They involve about 7% of all femur fractures, with bimodal age distribution, commonly occur during high-velocity trauma of motor vehicle accidents in the younger group of patients and are frequently associated with other skeletal injuries. The treatment of distal femoral fractures has evolved from conservative treatment to more aggressive operative treatment. The aim is to achieve and maintain a good reduction of the joint to allow early active mobilisation, thus minimising the joint stiffness and severe muscular atrophy encountered in the conservative treatment.

**Materials and methods:**

This is a retrospective study of 25 patients with distal femur fracture with intra-articular extension treated with open reduction and internal fixation with DFLP, admitted at our institute between 2016 to 2019, with a minimum follow-up of six months.

**Results:**

In our study, 19 (76%) patients had excellent to good results. Three (12%) patients had fair outcomes, and three (12%) patients had poor outcomes according to Neer’s score. The average time for bone union in closed fractures was earlier (4.25 months) than open fractures, averaging 5.86 months. The outcome was almost similar between closed and open fractures. There were 2 (8%) cases of infection in the early post-operative period, 7 (12%) patients suffered from knee stiffness, and there were 3 (12%) cases with a pre-operative bone loss that required bone grafting.

**Conclusion:**

Management of complex intra-articular distal femur fracture has always been a challenge. Anatomical reduction of articular fragments and rigid fixation of these fractures are a must. DFLP provides angular stability with multiple options to secure fixation of both metaphyseal and articular fragments with the restoration of the joint congruity, limb length, alignment and rotation, allowing early mobilisation and aggressive physiotherapy without loss of fixation, resulting in gratifying functional outcome and low complication rate.

## Introduction

Fractures affecting the distal femur with intra-articular extension are complex injuries that pose a challenge to every orthopaedic surgeon. These serious injuries produce significant disabilities. The incidence of complex distal femur fractures is increasing due to an increase in high-velocity road traffic accidents. Fractures of the distal femur account for 0.4% of all fractures. They involve about 7% of all femur fractures^1,2^ with a bimodal age distribution and commonly occurring during high-velocity trauma related to motor vehicle accidents in the younger group of patients and are frequently associated with other skeletal injuries. In contrast, elderly patients with severe osteopenia might sustain isolated distal femur fractures from trivial trauma.

The treatment of distal femoral fracture has evolved from non-operative, conservative treatment to more aggressive operative treatment. The aim is to achieve and maintain a good reduction of the joint and allow early active mobilisation, thus minimising the joint stiffness and severe muscular atrophy encountered in the conservative treatment^3,4,5^. Numerous devices such as angle blade plate, dynamic condylar screw, less invasive stabilisation system (LISS), intramedullary nails, and distal femoral locking plate (DFLP) have been proposed for the treatment of the distal femur fracture. We evaluated results of these fractures treated with open reduction and internal fixation, mostly with DFLP (Distal femoral locking plate)

## Materials and Methods

This is a retrospective study of 25 patients with distal femur fracture with intra-articular extension treated with open reduction and internal fixation with the same type of DFLP. They were admitted to our institute between 2016 to 2019 with a minimum follow-up of six months.

Patients above the age of 18 years having AO/OTA type 33. C1/ C2/C3 fractures were included in the study. Patients with AO type A and B fractures, Grade III-C open fractures, and pathological fractures were excluded from this study.

After stabilising the patient hemodynamically, standard antero-posterior and lateral radiographs were done. A CT scan of the affected knee was done for a better understanding of the fracture pattern. Fractures were then classified according to the AO classification. Lower tibial skeletal traction and above knee slab were applied, and the limb was elevated on a Bohler Braun splint. If the fracture was open, antibiotics and tetanus prophylaxis were given, debridement of the wound and external fixation were done primarily, and definitive fixation was done once the wound improved. In the case of a closed fracture, the patient was electively posted for surgery after all the routine investigations were done and anaesthetic clearance was obtained. The surgeries were done by three surgeons.

All the patients were operated on under spinal anaesthesia. Patients were positioned on the radiolucent table allowing visualisation of both AP and lateral views under the C-arm. Patients were positioned in a supine position with knee flexed at 30° by putting a sterile bolster below the knee as this relaxed the gastrocnemius and also facilitated exposure and reduction. A small sandbag was placed just behind the buttock to prevent external rotation of the limb. In complex fractures, preparation of both the limbs were done to achieve correct adjustment and comparison of length and rotation.

**Fig 1:**
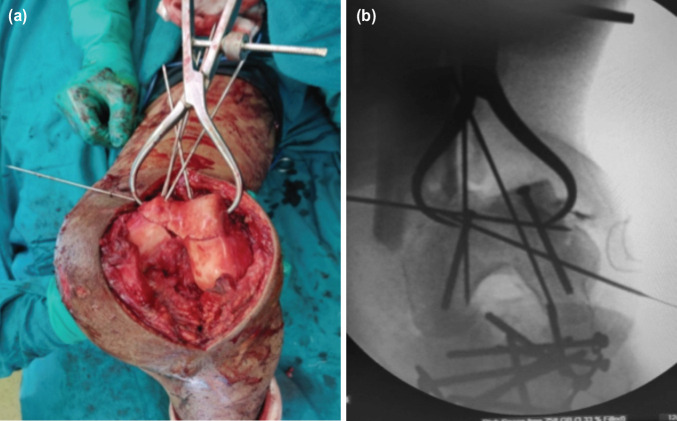
(a) Provisional fixation, (b) C arm image of the same inter-condylar fragment with K wires and clamp and screws.

The lateral parapatellar approach was preferred for all the fractures as it provided good exposure of the joint. After marking the tibial tuberosity and patella, a midline or preferably slightly lateral to midline incision from a point 5cm above the superior pole of the patella to below the level of tibial tuberosity was made. Medial and lateral skin flaps were developed to expose the quadriceps tendon, the lateral border of the patella and the lateral border of the patellar tendon. After exposing the lateral aspect of the patella, a full-thickness, longitudinal incision was made through the lateral para-patellar retinaculum and quadriceps tendon, beginning slightly lateral to the midline and curved to the lateral aspect of the patella leaving 8mm to 10mm cuff of the patellar retinaculum on the lateral aspect of the patella. By knee flexion and medial traction on the extensor mechanism, the patella was dislocated medially. Anatomical reduction of intra-articular fragments was done (Fig. 1).

Joint reconstruction was then performed with direct reduction. Each condyle was fully assessed first for smaller fracture fragments to restore each condyle anatomically. Large coronal fracture fragments are best treated with countersunk 3.5 to 4.5mm lag-type screws. The articular block with the shaft was aligned, and a lateral submuscular tunnel along the shaft was created to slide the plate through it.

After fixing the articular block by distal screws of the plate, the plate was fixed in the proximal part by bridging the fracture site respecting the fracture hematoma and achieving relative stability allowing micromotion at the fracture site after achieving satisfactory limb length, with sagittal and rotational reduction at the fracture site.

If there was severe metaphyseal comminution, then additional medial side plating was done (five patients). From time to time, in different phases of operative steps, plate fixation, reduction and screw placement were verified under fluoroscopy. A wash was given in the operative field with normal saline, and the wound was then closed in layers over a drain.

An immediate post-operative radiograph was done. An above knee posterior splint was applied and elevated over a Bohler Braun splint. Parenteral antibiotics were given till the 2nd post-operative day. After that, oral antibiotics were given till suture removal. Sutures were removed between the 12th to 15th post-operative days. Knee mobilisation was started once the patient was pain-free depending upon the fixation achieved of the fracture comminution.

Post-operative rehabilitation was started according to the stability of fixation, which was assessed intra-operatively. The range of motion was started on the 2nd day, emphasising extension, normal patella mobility, and the control of oedema, and pain.

Isometric quadriceps strengthening and hamstring stretching exercises were encouraged. Partial weight-bearing was started after 8 weeks, and full weight-bearing was allowed after radiological evidence of healing (12-16 weeks).

**Fig 2:**
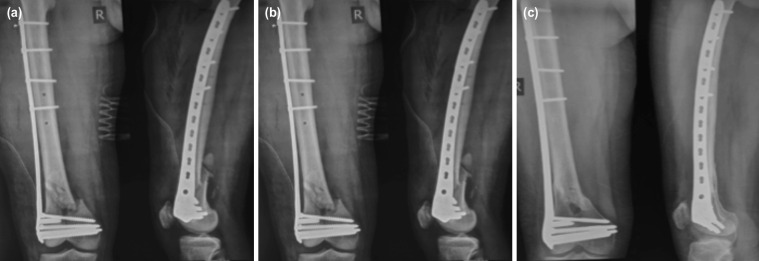
(a) Pre-operative radiograph (b) Immediate post-operative radiograph, (c) six months post-operative radiograph.

Regular follow-up was done at a monthly interval until fracture union, and three monthly after that (Fig. 2). In each visit, functional outcome was analysed, and a digital radiograph of the knee with distal femur was taken to assess the union of the fracture. In addition, the functional outcome of all the patients was analysed using Neer’s scoring system6, and the complications were documented.

**Fig 3:**
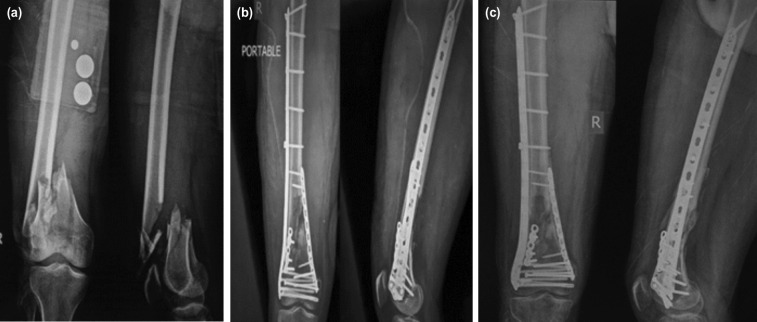
(a) Pre-operative radiograph (b) Immediate post-operative radiograph, (c) six months post-operative radiograph.

## Results

In our study of 25 patients, the youngest patient was 19 years old, and the oldest patient was 72 years old. The average age was 42.5 years. The highest incidence of fracture was in the age group of 18-47 years, 72% with 18 patients., Males were more affected, 80% with 20 patients, as compared to females, 20% with five patients.

The right side was more commonly affected, 64% with 16 patients, than the left side, 36% with 9 patients. The mode of injury was RTA in 22 patients (88%) whereas 3 patients (12%) were injured following a fall.

In our study 8 patients (32%) had closed fractures, whereas 17 patients (68%) had open fractures. The most common fracture pattern was AO type C3 in 11 patients (44%), followed by AO type C1 in 8 patients (32%), and AO type C2 in 6 patients (24%). Twenty-two patients (88%) were operated on within 8 days of admission, whereas 3 patients (12%) had more than 8 days of delay in definitive fixation due to the poor soft tissue condition. Six patients (24%) were discharged within seven days of admission. Nineteen patients (28%) were discharged after 7 days due to open fracture and associated injuries.

In 8 patients (32%), the bony union was seen at 4 and 5 months and in 6 patients (24%), the bony union was seen at 6 months, whereas in 3 patients (12%), the bony union was seen at more than 6 months due to bone loss and they required bone grafting. The average union time was 5.47 months. The average time for bone union in closed fractures was earlier, at 4.25 months, as compared to open fractures, averaging 5.86 months (Table I). There was no malalignment.

**Table I:** Bone union time

Time (in months)	Number/percent
4 months	8 (32%)
5 months	8 (32%)
6 months	6 (24%)
More than 6 months	3 (12%)
Total	25

There were 2 (8%) cases of infection in the early post-operative period in our study. The first case developed an infection before definitive fixation and was managed by debridement and regular wound management. The second case developed a small local site infection, which was managed by debridement and regular dressing. Culture sensitivity was done in both cases, and parenteral antibiotics were started accordingly, and the infection was cured. Seven (12%) patients suffered from knee stiffness, with less than 90° of flexion. Of these patients, five (20%) patients were AO Type C3 fracture, one (4%) patient was AO Type C2, and one (4%) patient was AO Type C1.

There were 3 (12%) cases with pre-operative bone loss of 2 to 3cms which required bone grafting. Out of these, two patients were grafted at three months, and the third patient was lost to follow-up for one year, so bone grafting was done at one year. All these fractures were united.

In our study, of the total of 17 open grade fractures, only 3 (17%) cases had excellent results; 10 (59%) cases had a good outcome, and 2 cases (12%) had a fair outcome, and 2 (12%) cases had a poor outcome. Two (25%) cases of the 8 closed fractures had an excellent outcome, and 4 (50%) cases had a good outcome, and 1 (12.5%) case had a fair outcome, and 1 (12.5%) case had a poor outcome. In our study, the outcome was almost similar between closed and open fractures (Table II).

**Table II:** Result in open and closed fractures

Type of Fracture		No. of patients	Excellent	Good	Fair	Poor
Open	OG1	1	0	1	0	0
	OG2	6	2	2	1	1
	OG3	10	1	7	1	1
	Total	17	3	10	2	2
Closed		8	2	4	1	1
Total		25	5	14	3	3

In our study, AO type C1 had excellent functional outcome in 1 (4%) patient and good outcome in 6 (24%) patients and a fair outcome in 1 (4%) patient. AO type C2 had 3 (12%) cases with excellent outcomes, 2 (8%) cases with good outcomes and 1 (4%) case with a fair outcome. AO type C3 had an excellent outcome in 1 (4%) patient, good outcomes in 6 (24%) patients and a fair outcome in 1 (4%) patient and poor outcomes in 3 (12%) patients. The fair and poor outcomes were more common in Type C3 fracture (Table III). Nineteen (76%) patients had excellent to good results. Three (12%) patients had fair outcomes, and 3 (12%) patients had poor outcomes according to the NEER score.

**Table III:** Results according to classification of the fracture

Type of Fracture	Number	Percentage	Excellent	Good	Fair	Poor
C1	8	32%	1	6	1	0
C2	6	24%	3	2	1	0
C3	11	44%	1	6	1	3
Total	25	100%	5	14	3	3

## Discussion

The principle and goal of treatment of complex intra-articular distal femur fractures is the precise anatomical reduction and fixation of the articular surface with absolute stability, stabilisation of the meta-diaphyseal component and restoration of length alignment and rotation following the principles of relative stability and allowing early mobilisation. ORIF is the most reliable method to ensure articular surface restoration, with respect to coronal, axial and sagittal plane alignment.

The distal thigh and knee area include bone, articular surface, and soft tissue complex where the erroneous plate and/or screw placement may cause a number of problems with considerable consequences. Plate application has become less complex with the evolution of better implants that are potentially more effective with options for screws to be standard or locked, cannulated or non-cannulated, bi- or uni-cortical, and variable angled plates. Extensive as well as minimally invasive approaches and anatomically contoured plates help in better fracture fixation.

The DFLP system offers a number of advantages in fracture fixation, combining angular stability through the use of locking screws with traditional fixation techniques. Rademakers *et al*^7^, in their study on 67 patients at one-year follow-up, had a mean knee range of motion of 111° with good to excellent results in 84% patients according to Neer’s score. The study concluded that surgical treatment of mono and bicondylar femoral fractures shows a good long-term result after open reduction and internal fixation, and knee function increases through time, though the ROM does not increase after one year.

Virk JS *et al*^8^, in their study of 25 patients with a mean follow-up duration of 24 weeks, 20 patients had good to excellent results and fair results in five patients according to the NEER scoring system. The average duration of the union in all the cases was 5.47 months (21-22 weeks) in our study. In closed fractures, the average duration of union was 4.25 months (16-18 weeks), with none in the closed group requiring secondary bone grafting. For open fractures, the average duration of union increased to 5.86 months (22-23 weeks) thus, making open fractures a risk factor for the delayed union. The same concern was voiced by Ricci *et al*^9^, in their study on open fractures, as risk factors for longer duration of union. One risk factor within the surgeon's control affecting fracture union and possible cause of failure was the use of shorter plate length when spanning comminuted fractures.

Complications related to slow healing, including delayed union, are frequently encountered and are ongoing problems in managing these fractures. In the current study, 3 (12%) patients had primary bone loss, which healed after bone grafting. No cases of non-union were encountered in our study. Multiple reasons influence union rates: the coexisting patient morbidity; comminution at the fracture site; bone loss; and initial damage to the surrounding soft tissue.

Infection was found in 2 cases (8%) in our study, which responded to debridement and parenteral antibiotics and regular wound management. Culture sensitivity was done in both cases, and parenteral antibiotics were started accordingly. All the patients that developed infection had open fractures.

Hoffmann *et al*^10^ also reported a similar rate of infection, 7.2% in his study, of which 1 was superficial, and 8 were deep infections requiring secondary surgery. All the patients that developed infection, superficial or deep, were from the open fracture group.

Knee stiffness was the most common late complication in our study, affecting 7 patients (28%). Of these patients, 5 (20%) had AO Type C3 fracture, 1 (4%) had AO Type C2 fracture and 1 (4%) had AO Type C1 fracture. This complication invariably results from damage to the quadriceps mechanism and joint surface because of the initial trauma or surgical exposure for fixation or both. Quadriceps scarring with or without arthrofibrosis of the knee or patella-femoral joint is thought to restrict knee movement. These effects are greatly magnified by immobilisation after the fracture or internal fixation. Degenerative osteoarthritis of the knee also affects knee movement. Mal-alignment of 10° is likely to affect knee mechanics and gait. Increased varus or valgus may lead to overloading of the joint and subsequent arthrosis of the medial or lateral compartment, respectively.

The outcome of distal femoral fractures, like other major injuries, not only depends on bony reconstruction but also soft tissue management. Open wounds require thorough debridement and wash for prevention of complications and achievement of better knee functions. Early stable internal fixation of the fracture with meticulous soft tissue handling and early mobilisation of the knee joint maximise the chance for an optimal outcome after a distal femur fracture.

Although the DFLP system offers a number of advantages in fracture management, successful outcome requires careful pre-operative planning, precise application of biomechanical principles, and the use of the appropriate plate and screws combined with good surgical technique.

## Conclusion

Management of complex intra-articular distal femur fracture (AO type C) is always a challenge for the orthopaedic fraternity. Anatomical reduction of articular fragments and rigid fixation of these fractures are musts. DFLP provides angular stability and provides multiple options to secure fixation of both metaphyseal and articular fragments with restoration of the joint congruity, limb length, alignment and rotation, allowing early mobilisation and aggressive physiotherapy without loss of fixation, resulting in gratifying functional outcome and low complication rate.
